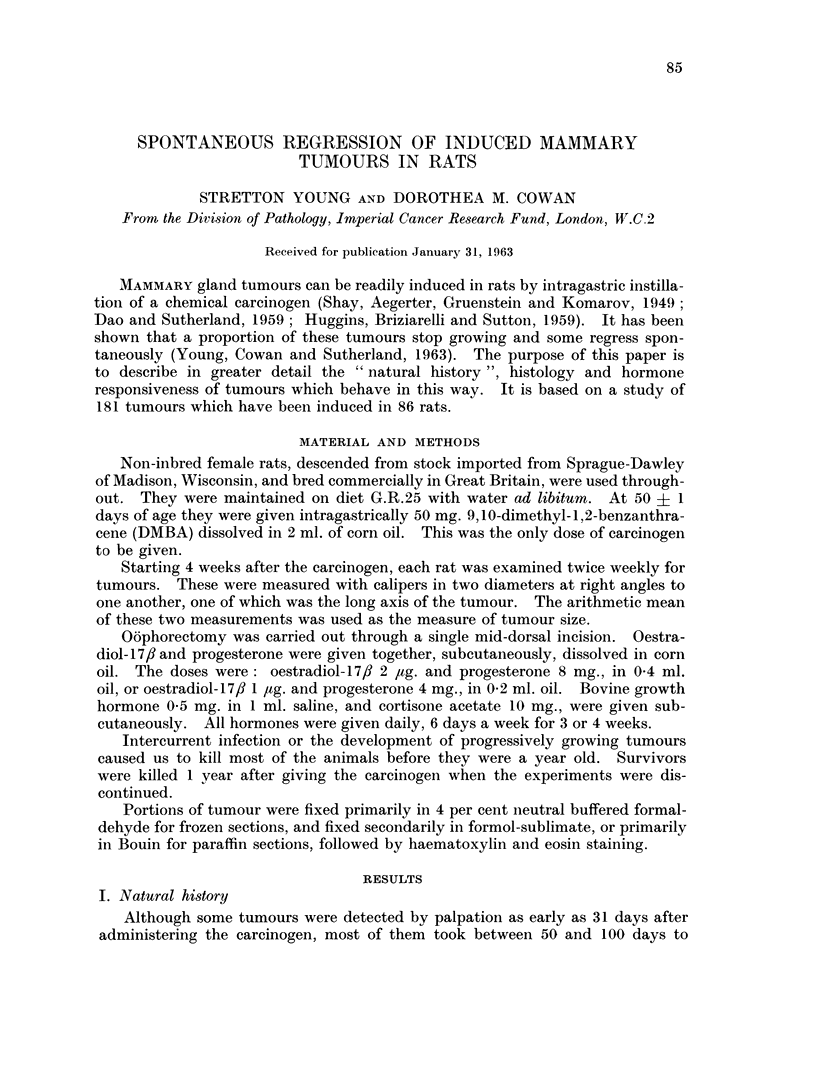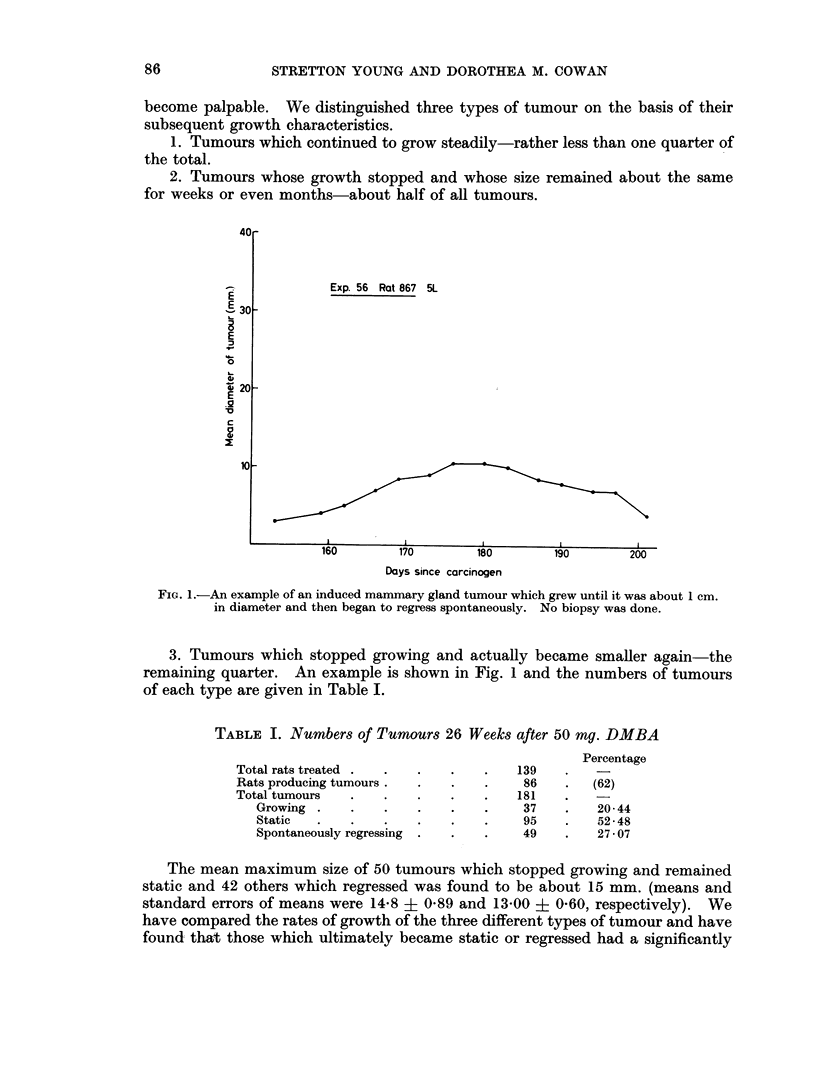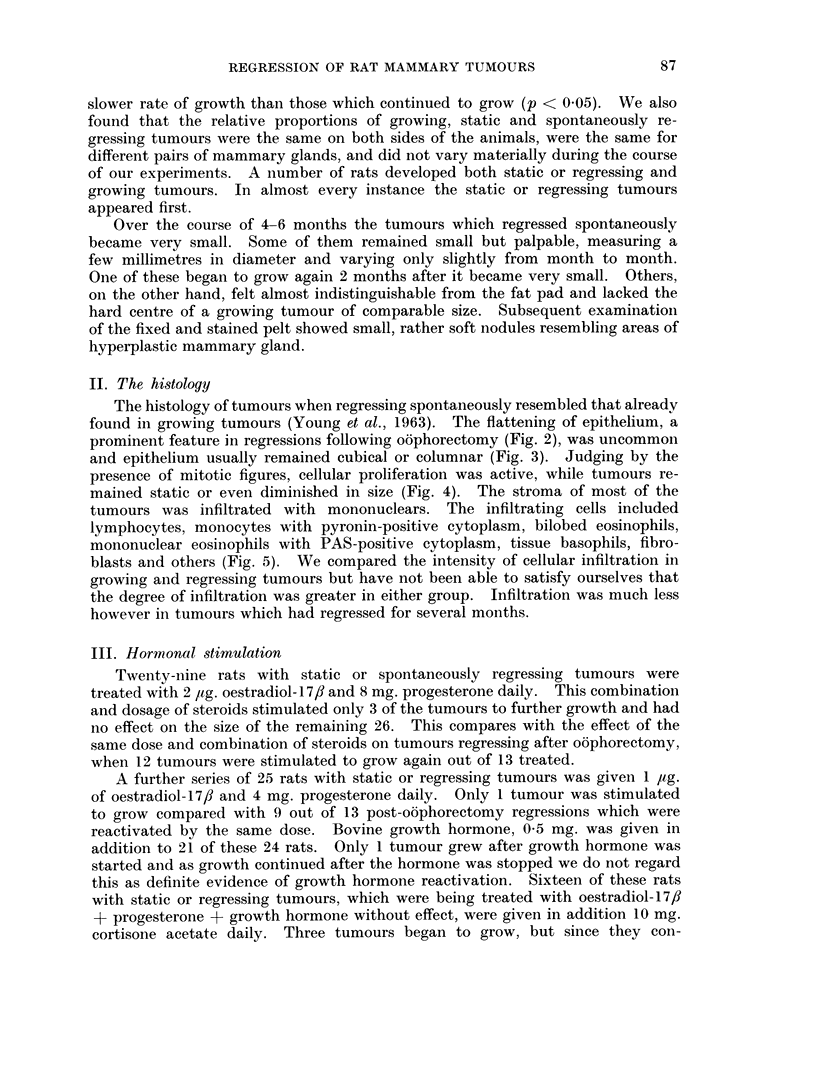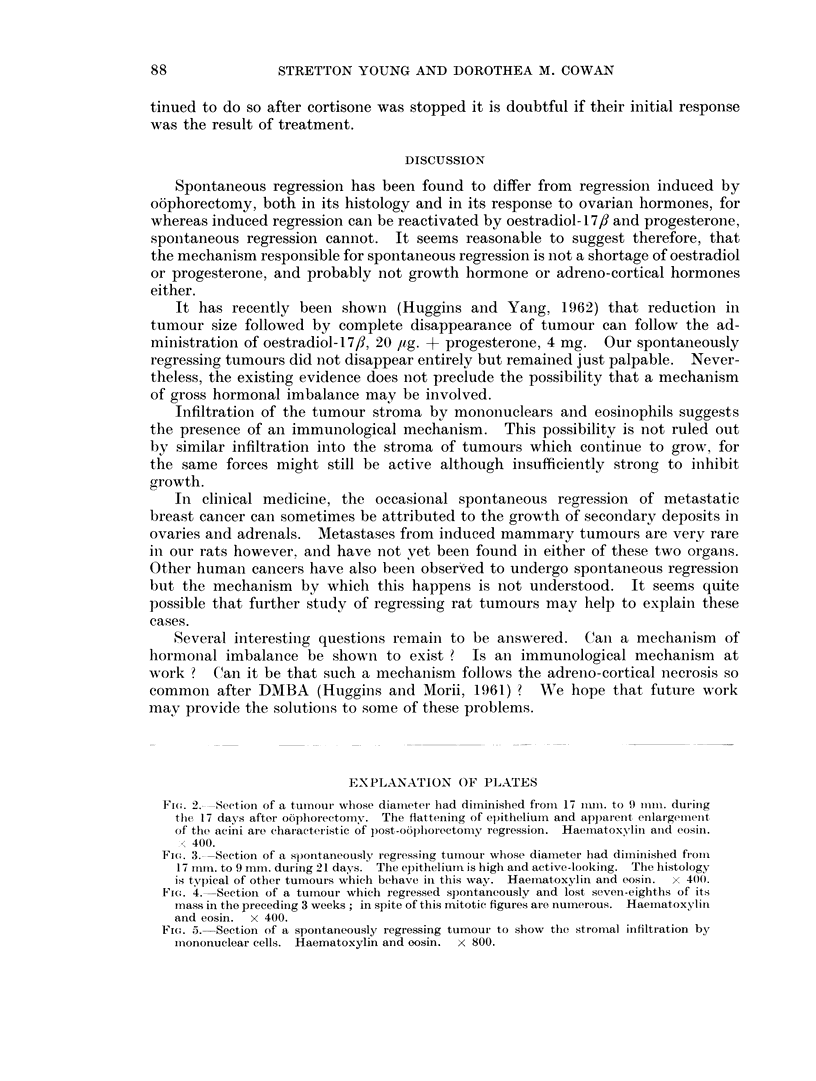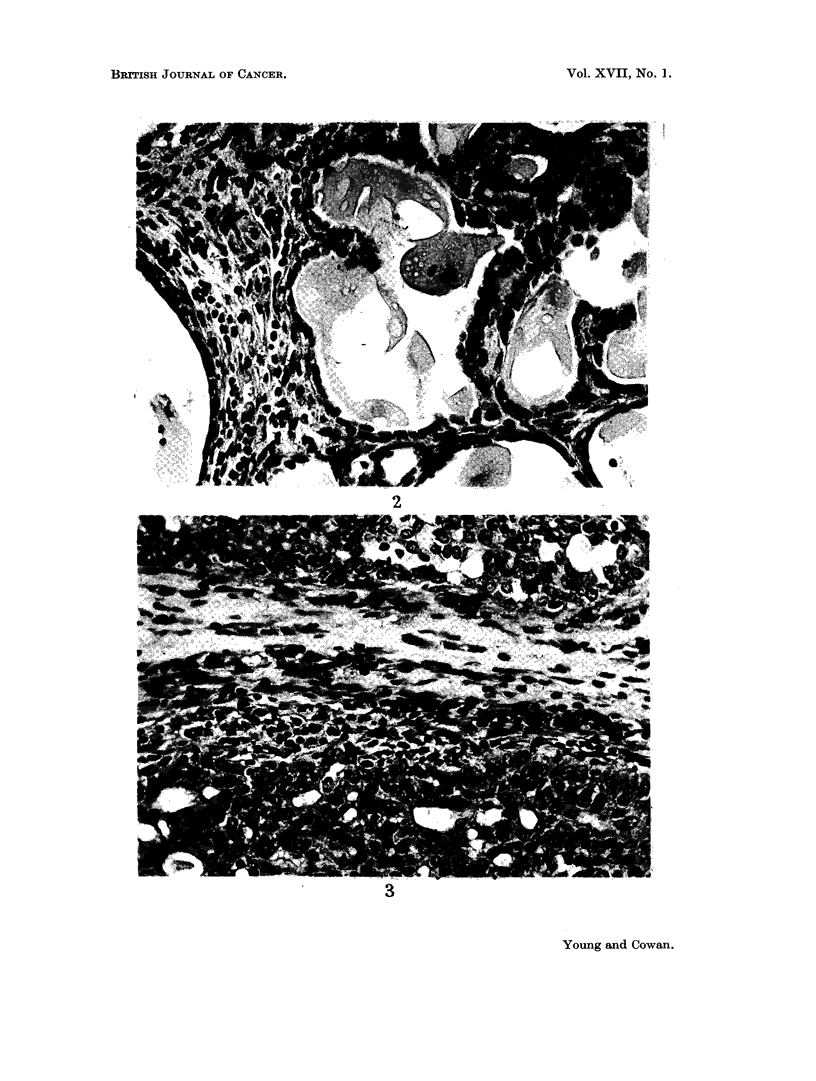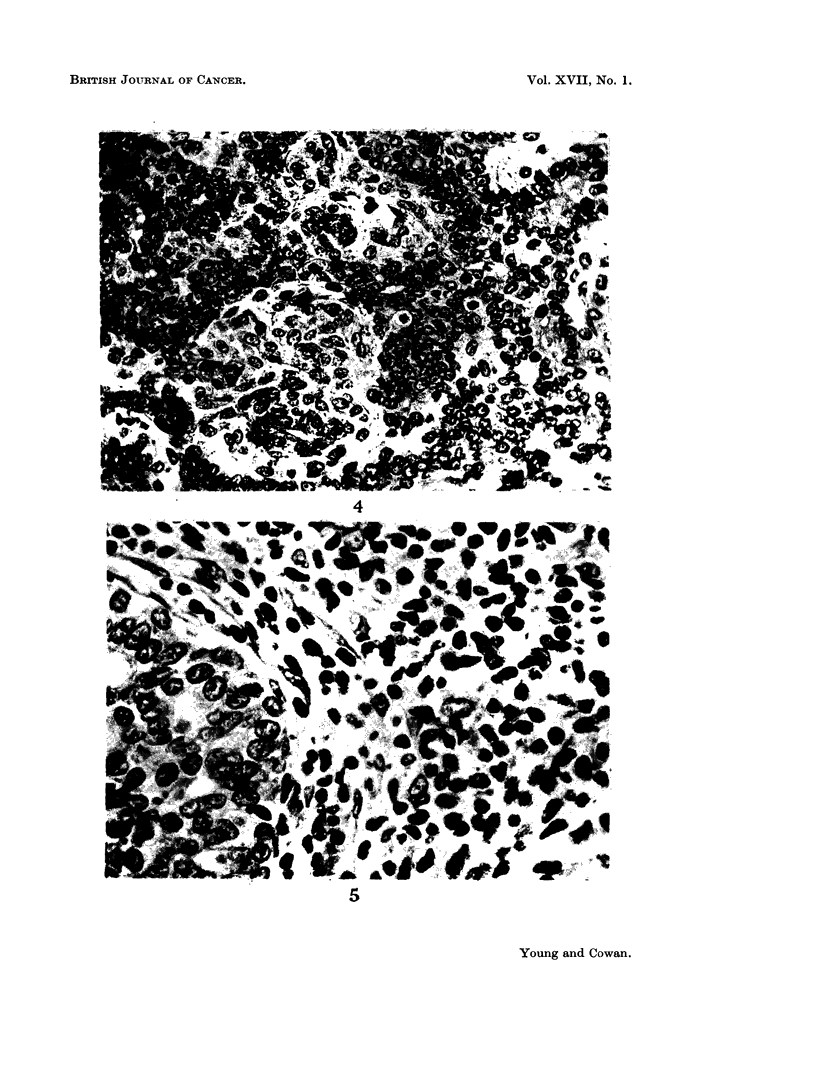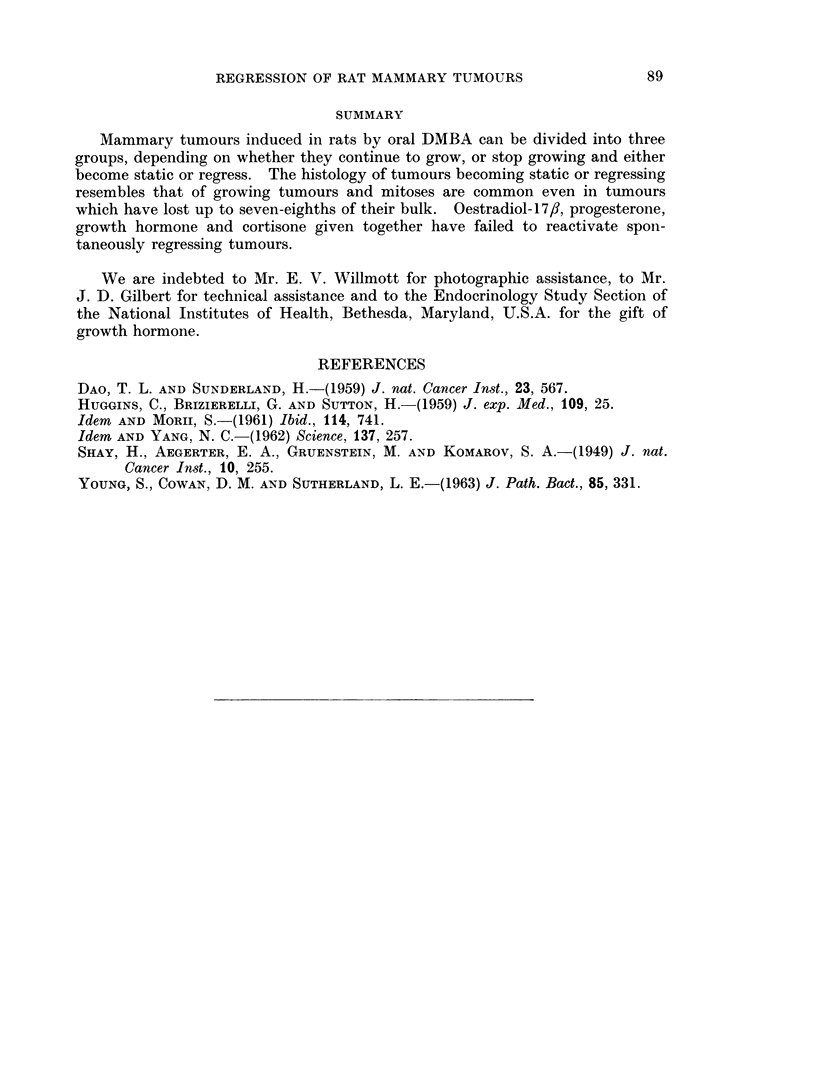# Spontaneous Regression of Induced Mammary Tumours in Rats

**DOI:** 10.1038/bjc.1963.13

**Published:** 1963-03

**Authors:** Stretton Young, Dorothea M. Cowan

## Abstract

**Images:**


### SPONTANEOUS REGRESSION OF INDUCED MAMMARY TUMOURS IN RATS

STRETTON YOUNG AND DOROTHEA M. COWAN
*From the Division of Pathology, Imperial Cancer Research Fund, London, W.C.2*

Received for publication January 31, 1963

MAMMARY gland tumours can be readily induced in rats by intragastric instillation of a chemical carcinogen (Shay, Aegerter, Gruenstein and Komarov, 1949; Dao and Sutherland, 1959; Huggins, Briziarelli and Sutton, 1959). It has been shown that a proportion of these tumours stop growing and some regress spontaneously (Young, Cowan and Sutherland, 1963). The purpose of this paper is to describe in greater detail the "natural history", histology and hormone responsiveness of tumours which behave in this way. It is based on a study of 181 tumours which have been induced in 86 rats.

### MATERIAL AND METHODS

Non-inbred female rats, descended from stock imported from Sprague-Dawley of Madison, Wisconsin, and bred commercially in Great Britain, were used throughout. They were maintained on diet G.R.25 with water *ad libitum*. At $50 \pm 1$ days of age they were given intragastrically 50 mg. 9,10-dimethyl-1,2-benzanthracene (DMBA) dissolved in 2 ml. of corn oil. This was the only dose of carcinogen to be given.

Starting 4 weeks after the carcinogen, each rat was examined twice weekly for tumours. These were measured with calipers in two diameters at right angles to one another, one of which was the long axis of the tumour. The arithmetic mean of these two measurements was used as the measure of tumour size.

Oöphorectomy was carried out through a single mid-dorsal incision. Oestradiol-17$\beta$ and progesterone were given together, subcutaneously, dissolved in corn oil. The doses were: oestradiol-17$\beta$ 2 $\mu$g. and progesterone 8 mg., in 0·4 ml. oil, or oestradiol-17$\beta$ 1 $\mu$g. and progesterone 4 mg., in 0·2 ml. oil. Bovine growth hormone 0·5 mg. in 1 ml. saline, and cortisone acetate 10 mg., were given subcutaneously. All hormones were given daily, 6 days a week for 3 or 4 weeks.

Intercurrent infection or the development of progressively growing tumours caused us to kill most of the animals before they were a year old. Survivors were killed 1 year after giving the carcinogen when the experiments were discontinued.

Portions of tumour were fixed primarily in 4 per cent neutral buffered formaldehyde for frozen sections, and fixed secondarily in formol-sublimate, or primarily in Bouin for paraffin sections, followed by haematoxylin and eosin staining.

### RESULTS

### I. *Natural history*

Although some tumours were detected by palpation as early as 31 days after administering the carcinogen, most of them took between 50 and 100 days to become palpable. We distinguished three types of tumour on the basis of their subsequent growth characteristics.

1. Tumours which continued to grow steadily—rather less than one quarter of the total.

2. Tumours whose growth stopped and whose size remained about the same for weeks or even months—about half of all tumours.

FIG. 1.—An example of an induced mammary gland tumour which grew until it was about 1 cm. in diameter and then began to regress spontaneously. No biopsy was done.

3. Tumours which stopped growing and actually became smaller again—the remaining quarter. An example is shown in Fig. 1 and the numbers of tumours of each type are given in Table I.

TABLE I. *Numbers of Tumours* 26 *Weeks after* 50 *mg. DMBA*

| | | Percentage |
|---|---|---|
| Total rats treated | 139 | — |
| Rats producing tumours | 86 | (62) |
| Total tumours | 181 | — |
| Growing | 37 | 20·44 |
| Static | 95 | 52·48 |
| Spontaneously regressing | 49 | 27·07 |

The mean maximum size of 50 tumours which stopped growing and remained static and 42 others which regressed was found to be about 15 mm. (means and standard errors of means were 14·8 ± 0·89 and 13·00 ± 0·60, respectively). We have compared the rates of growth of the three different types of tumour and have found that those which ultimately became static or regressed had a significantly slower rate of growth than those which continued to grow ($p < 0{\cdot}05$). We also found that the relative proportions of growing, static and spontaneously regressing tumours were the same on both sides of the animals, were the same for different pairs of mammary glands, and did not vary materially during the course of our experiments. A number of rats developed both static or regressing and growing tumours. In almost every instance the static or regressing tumours appeared first.

Over the course of 4–6 months the tumours which regressed spontaneously became very small. Some of them remained small but palpable, measuring a few millimetres in diameter and varying only slightly from month to month. One of these began to grow again 2 months after it became very small. Others, on the other hand, felt almost indistinguishable from the fat pad and lacked the hard centre of a growing tumour of comparable size. Subsequent examination of the fixed and stained pelt showed small, rather soft nodules resembling areas of hyperplastic mammary gland.

### II. *The histology*

The histology of tumours when regressing spontaneously resembled that already found in growing tumours (Young *et al.*, 1963). The flattening of epithelium, a prominent feature in regressions following oöphorectomy (Fig. 2), was uncommon and epithelium usually remained cubical or columnar (Fig. 3). Judging by the presence of mitotic figures, cellular proliferation was active, while tumours remained static or even diminished in size (Fig. 4). The stroma of most of the tumours was infiltrated with mononuclears. The infiltrating cells included lymphocytes, monocytes with pyronin-positive cytoplasm, bilobed eosinophils, mononuclear eosinophils with PAS-positive cytoplasm, tissue basophils, fibroblasts and others (Fig. 5). We compared the intensity of cellular infiltration in growing and regressing tumours but have not been able to satisfy ourselves that the degree of infiltration was greater in either group. Infiltration was much less however in tumours which had regressed for several months.

### III. *Hormonal stimulation*

Twenty-nine rats with static or spontaneously regressing tumours were treated with 2 $\mu$g. oestradiol-17$\beta$ and 8 mg. progesterone daily. This combination and dosage of steroids stimulated only 3 of the tumours to further growth and had no effect on the size of the remaining 26. This compares with the effect of the same dose and combination of steroids on tumours regressing after oöphorectomy, when 12 tumours were stimulated to grow again out of 13 treated.

A further series of 25 rats with static or regressing tumours was given 1 $\mu$g. of oestradiol-17$\beta$ and 4 mg. progesterone daily. Only 1 tumour was stimulated to grow compared with 9 out of 13 post-oöphorectomy regressions which were reactivated by the same dose. Bovine growth hormone, 0·5 mg. was given in addition to 21 of these 24 rats. Only 1 tumour grew after growth hormone was started and as growth continued after the hormone was stopped we do not regard this as definite evidence of growth hormone reactivation. Sixteen of these rats with static or regressing tumours, which were being treated with oestradiol-17$\beta$ + progesterone + growth hormone without effect, were given in addition 10 mg. cortisone acetate daily. Three tumours began to grow, but since they continued to do so after cortisone was stopped it is doubtful if their initial response was the result of treatment.

### DISCUSSION

Spontaneous regression has been found to differ from regression induced by oöphorectomy, both in its histology and in its response to ovarian hormones, for whereas induced regression can be reactivated by oestradiol-17$\beta$ and progesterone, spontaneous regression cannot. It seems reasonable to suggest therefore, that the mechanism responsible for spontaneous regression is not a shortage of oestradiol or progesterone, and probably not growth hormone or adreno-cortical hormones either.

It has recently been shown (Huggins and Yang, 1962) that reduction in tumour size followed by complete disappearance of tumour can follow the administration of oestradiol-17$\beta$, 20 $\mu$g. + progesterone, 4 mg. Our spontaneously regressing tumours did not disappear entirely but remained just palpable. Nevertheless, the existing evidence does not preclude the possibility that a mechanism of gross hormonal imbalance may be involved.

Infiltration of the tumour stroma by mononuclears and eosinophils suggests the presence of an immunological mechanism. This possibility is not ruled out by similar infiltration into the stroma of tumours which continue to grow, for the same forces might still be active although insufficiently strong to inhibit growth.

In clinical medicine, the occasional spontaneous regression of metastatic breast cancer can sometimes be attributed to the growth of secondary deposits in ovaries and adrenals. Metastases from induced mammary tumours are very rare in our rats however, and have not yet been found in either of these two organs. Other human cancers have also been observed to undergo spontaneous regression but the mechanism by which this happens is not understood. It seems quite possible that further study of regressing rat tumours may help to explain these cases.

Several interesting questions remain to be answered. Can a mechanism of hormonal imbalance be shown to exist ? Is an immunological mechanism at work ? Can it be that such a mechanism follows the adreno-cortical necrosis so common after DMBA (Huggins and Morii, 1961) ? We hope that future work may provide the solutions to some of these problems.

### EXPLANATION OF PLATES

FIG. 2.—Section of a tumour whose diameter had diminished from 17 mm. to 9 mm. during the 17 days after oöphorectomy. The flattening of epithelium and apparent enlargement of the acini are characteristic of post-oöphorectomy regression. Haematoxylin and eosin. × 400.

FIG. 3.—Section of a spontaneously regressing tumour whose diameter had diminished from 17 mm. to 9 mm. during 21 days. The epithelium is high and active-looking. The histology is typical of other tumours which behave in this way. Haematoxylin and eosin. × 400.

FIG. 4.—Section of a tumour which regressed spontaneously and lost seven-eighths of its mass in the preceding 3 weeks ; in spite of this mitotic figures are numerous. Haematoxylin and eosin. × 400.

FIG. 5.—Section of a spontaneously regressing tumour to show the stromal infiltration by mononuclear cells. Haematoxylin and eosin. × 800.

2

3

4

5

SUMMARY

Mammary tumours induced in rats by oral DMBA can be divided into three groups, depending on whether they continue to grow, or stop growing and either become static or regress. The histology of tumours becoming static or regressing resembles that of growing tumours and mitoses are common even in tumours which have lost up to seven-eighths of their bulk. Oestradiol-17$\beta$, progesterone, growth hormone and cortisone given together have failed to reactivate spontaneously regressing tumours.

We are indebted to Mr. E. V. Willmott for photographic assistance, to Mr. J. D. Gilbert for technical assistance and to the Endocrinology Study Section of the National Institutes of Health, Bethesda, Maryland, U.S.A. for the gift of growth hormone.

### REFERENCES

DAO, T. L. AND SUNDERLAND, H.—(1959) *J. nat. Cancer Inst.*, **23**, 567.
HUGGINS, C., BRIZIERELLI, G. AND SUTTON, H.—(1959) *J. exp. Med.*, **109**, 25.
*Idem* AND MORII, S.—(1961) *Ibid.*, **114**, 741.
*Idem* AND YANG, N. C.—(1962) *Science*, **137**, 257.
SHAY, H., AEGERTER, E. A., GRUENSTEIN, M. AND KOMAROV, S. A.—(1949) *J. nat. Cancer Inst.*, **10**, 255.
YOUNG, S., COWAN, D. M. AND SUTHERLAND, L. E.—(1963) *J. Path. Bact.*, **85**, 331.